# Hierarchical regression analysis of physical fitness and BMI in primary school students in Sichuan Province

**DOI:** 10.3389/fmed.2026.1807499

**Published:** 2026-07-03

**Authors:** Defa Zhang, Wennan Zhao, Jingtao Wu, Jun Hu, Xinjuan Zhao

**Affiliations:** 1 School of Physical Education, Wuxi Taihu University, Wuxi, China; 2 School of Physical Education, Yanshan University, Qinhuangdao, Hebei, China; 3 School of Physical Education, Leshan Normal University, Leshan, China

**Keywords:** BMI, hierarchical regression analysis, physical fitness, primary school students, Sichuan Province

## Abstract

**Background:**

The increasing prevalence of childhood obesity has made Body Mass Index (BMI) a crucial indicator for assessing children’s physical health. Despite extensive research, the correlation between BMI and physical fitness among China’s ethnically diverse youth remains poorly understood. This study investigates the associations between BMI and physical fitness indicators among primary school students in Sichuan Province, with particular attention to differences by gender, ethnicity, and age.

**Methods:**

The study analyzed physical fitness data from 36,092 primary school students aged 7–12 in Sichuan Province. Multivariate analysis of variance, correlation analysis, and hierarchical multiple regression analysis were employed to examine the associations between BMI and physical fitness indicators. Because data on several potential confounders, including diet, socioeconomic status, physical activity, and sleep, were unavailable, these variables were not included in the adjusted models.

**Results:**

The study revealed significant differences in BMI across different ethnicities, genders, and age groups. Han students had an average BMI of 17.07, which was significantly higher than the Yi students’ average of 16.90 (*F* = 31.942, *p* < 0.001); boys had an average BMI of 17.18, significantly higher than girls’ average of 16.85 (*F* = 131.733, *p* < 0.001); BMI increased significantly with age, with the 12-year-old group having the highest average (*M* = 18.08). BMI was negatively correlated with 50-meter run performance (*r* = −0.266, *p* < 0.01) and lung capacity (*r* = −0.186, *p* < 0.01). Hierarchical regression analysis showed that several demographic variables, physical fitness indicators, and interaction terms were statistically associated with BMI; however, most effect sizes were small, and the final model explained only a modest proportion of BMI variance (*R*^2^ = 0.110).

**Conclusion:**

BMI in primary school students in Sichuan Province was significantly associated with gender, ethnicity, age, and multiple physical fitness indicators. However, because several potential confounding variables were not adjusted for, these findings should be interpreted cautiously as observational associations. The findings provide context-specific evidence that may inform future hypothesis-driven research and regionally targeted public health planning; however, given the generally small effect sizes, their practical significance at the individual level may be limited.

## 1 Introduction

The persistent rise in childhood obesity rates worldwide has made the physical health status of children and adolescents a focal point of public health research in many countries. Obesity not only has profound effects on children’s physiological health, but is also closely associated with a variety of chronic diseases, such as cardiovascular diseases and diabetes (1). In recent years, an increasing number of studies have focused on Body Mass Index (BMI) as an important indicator for measuring children’s physical health, with complex correlations existing between BMI and various physical fitness indicators (2). These physical fitness indicators, including lung capacity, running performance, and flexibility, are important components of children’s overall fitness assessment (3). However, due to differences in gender, ethnicity, age group, and socioeconomic background among children, the ways in which these factors are associated with BMI and physical health are not yet fully understood (4). Therefore, in-depth research on how these factors are associated with children’s physical health is crucial for developing precise health interventions and promoting the healthy growth of children.

Although extensive discussions have been conducted by scholars at home and abroad on the relationship between BMI and children’s physical health, most studies have concentrated on economically developed urban areas, neglecting the special groups in less developed regions in western China (5). Sichuan Province, as a multi-ethnic settlement area in southwestern China, has a unique geographical environment and socio-economic structure, making the study of children’s physical health in this region of significant academic and practical importance. Existing research has shown that urban–rural disparities in economic development, unequal distribution of educational resources (6), and differences in lifestyle habits may contribute to significant differences in children’s physical fitness and BMI (7). However, current research on the relationship between physical health and BMI among primary school students in Sichuan Province is still relatively limited, especially lacking in systematic analysis of the impact of different physical fitness indicators, ethnicities, genders, and grades on BMI. The research gap limits a comprehensive understanding of children’s health status in this region and hinders the formulation of effective health intervention policies.

To fill in this research gap, the present study utilizes survey data from a large sample of primary school students in Sichuan Province and employs hierarchical regression analysis with interaction terms to systematically examine the associations of different physical fitness indicators, ethnicity, gender, and age with BMI. In the present study, hierarchical regression refers to the stepwise entry of predictors and interaction terms into successive regression models, rather than multilevel modeling, hierarchical linear modeling, or cross-level/cross-layer analysis in the mixed-effects sense. By considering demographic characteristics and physical fitness indicators available in the dataset, the study aims to examine how these factors are associated with BMI levels among primary school students in Sichuan Province. Specifically, this study analyzes the correlation between BMI and various physical fitness indicators and discusses whether variables such as gender, ethnicity, and grade are associated with differences in these relationships. Conceptually, the study is informed by a developmental and contextual perspective, in which BMI and physical fitness may vary across age and may also differ by gender and ethnicity. Based on this perspective, the study addresses three questions: whether BMI differs across gender, ethnicity, and age groups; whether physical fitness indicators are associated with BMI; and whether these associations vary by gender and ethnicity. Accordingly, we hypothesized that BMI would be significantly associated with demographic characteristics and physical fitness indicators, and that some of these associations would differ by gender and ethnicity. This study provides empirical evidence on the associations between BMI and physical fitness among primary school students in Sichuan Province and may inform future public health interventions in similar underdeveloped regions.

## 2 Research subjects and methods

### 2.1 Research design and data sources

The data for this study was collected through field research conducted from March to July 2024 across various regions in Sichuan Province, located in the southwestern part of China. Participants were primary school students, and the study employed a multi-stage, stratified, and random cluster sampling method to select representative samples from urban, county, and rural schools across the province. The research areas included Chengdu, Leshan, Liangshan Prefecture, Aba Prefecture, Mianyang, and Neijiang, aiming to systematically analyze the impact of different environmental and socioeconomic factors on the physical health and BMI of primary school students.

As an important province in the southwestern region of China, Sichuan boasts rich geographical and cultural diversity. Chengdu, as the provincial capital, represents the administrative, economic, and cultural center of Sichuan with a higher level of development; Leshan and Mianyang represent relatively developed second-tier cities; Liangshan and Aba are mainly remote areas inhabited by ethnic minorities with relatively lagging economic and cultural development; Neijiang reflects the characteristics of typical county and rural areas. By surveying these different regions, the study can more comprehensively reveal the differences in physical health and BMI among primary school students in urban and rural areas and among ethnic minority regions within Sichuan Province.

In terms of sample selection, several primary schools were randomly selected from the lists of schools provided by the education bureaus of each region. Specifically, 58 schools were randomly selected in urban areas and 55 schools in county and rural areas. Subsequently, two to three classes from each grade of each school were randomly selected as the subjects of the survey, and all eligible students were included in the research sample.

Regarding the inclusion and exclusion criteria, the study included all primary school students aged between 7 and 12 years old, and required all participants and their guardians to provide written informed consent. Students with severe heart, liver, or kidney diseases, as well as those with any conditions that might affect normal physical health assessment, were excluded from the study. Given that the study sample included predominantly Han and Yi students, with limited sample sizes from other ethnic groups, this analysis focused specifically on the Han and Yi populations to ensure statistical power and estimation stability, thereby reducing potential bias in data interpretation. The ethnicity-based analyses reported throughout this manuscript were conducted consistently for the Han and Yi groups only.

The study had obtained ethical approval from the Academic Committee of Leshan Normal University before implementation, with the ethical approval number being 202403021. The sample was highly representative, covering primary school students from various socioeconomic backgrounds and geographical environments in Sichuan Province. Through hierarchical regression analysis, the study aimed to examine how demographic variables and physical fitness indicators were associated with BMI among primary school students, providing reference information for child health intervention planning in Sichuan Province and other similar regions.

### 2.2 Measurement tools and methods

This study aimed to comprehensively assess the physical fitness status of primary school students in Sichuan Province and explore its association with BMI. The following details the measurement tools and specific methods used in the study. All measurements strictly followed the national standards for student physical health (GB/T 16432-2020) and related specifications (8).

#### 2.2.1 Measurement tools

The measurement tools included: a stadiometer for precise measurement of students’ height with a precision of 0.1 cm; a scale for measuring body weight with a precision of 0.1 kg; BMI calculation was completed using the formula BMI = weight (kg)/height (m)^2^ to assess students’ weight status; a spirometer for measuring students’ lung capacity in milliliters; a stopwatch for recording the time of the 50-meter run and the 50-meter shuttle run with a precision of 0.01 s; a tape measure and a flexible ruler for measuring the flexibility of the sit-and-reach test with a precision of 0.1 cm; and a counter for recording the number of jumps and sit-ups completed in 1 min.

#### 2.2.2 Measurement methods

In accordance with national standards, the study conducted strict measurements of the following physical health indicators (9). For height measurement, students stood barefoot with their backs straight, feet together, and the measurement was taken twice, with the average value being used. For weight measurement, students wore light clothing and were barefoot, and the measurement was taken twice, with the average value being used. BMI was calculated based on height and weight data. For lung capacity testing, students took a deep breath and then blew into the spirometer through their mouth, with the measurement taken twice and the highest value being used. The 50-meter run was conducted on a standard track, with students sprinting from the starting line to the finish line at full speed, and the completion time was recorded with a stopwatch, accurate to 0.01 s. The 50-meter shuttle run was also conducted on a standard track, with students completing 8 shuttle runs upon hearing the starting signal, and the completion time was recorded with a stopwatch. For the sit-and-reach test, students sat on the test board with legs straight and hands stacked, reaching forward as far as possible, and the farthest distance reached by the fingertips was recorded, with the measurement taken twice and the farthest value being used. The one-minute jump rope test and the one-minute sit-up test, respectively, recorded the number of jumps and sit-ups completed by students within 1 min.

#### 2.2.3 Data quality control

To ensure the accuracy and consistency of the measurement data, all measurements were carried out by specially trained measurers following a unified operational process. Each measurement used standardized measurement tools, and the measurement data were double-checked, with the average or highest value being taken. In addition, during the data entry and analysis process, researchers conducted strict screening for outliers and missing values to ensure the completeness and reliability of the data. All measurement methods and data processing were based on and referred to the national standards for student physical health to ensure the scientific nature and generalizability of the results.

### 2.3 Statistical methods

Data were analyzed using SPSS 26.0 and R 4.0.3. Descriptive statistics were used to summarize BMI and physical fitness indicators by ethnicity, gender, and age. Independent-samples *t* tests were used for two-group comparisons, and one-way analysis of variance (ANOVA) was used for comparisons across age groups. Levene’s test was conducted to assess homogeneity of variance, and Bonferroni correction was applied for *post hoc* pairwise comparisons when appropriate. In addition to *p* values, 95% confidence intervals (CIs) were reported where applicable.

Pearson correlation analysis was used to assess associations between BMI and physical fitness indicators. Hierarchical multiple regression analysis was performed to examine the incremental contribution of different predictor blocks to BMI. Demographic variables were entered in the first block, physical fitness indicators in the second block, and interaction terms in the third block. Both unstandardized coefficients (*B*) and standardized coefficients (*β*) were reported to facilitate interpretation and comparison across variables measured on different scales. Changes in *R*^2^ were used to evaluate the additional explanatory power of each model.

To reduce multicollinearity, interaction terms were mean-centered before analysis, and variance inflation factors (VIFs) were examined. Missing data were minimal (<3%). Because several potential confounders, including diet, socioeconomic status, physical activity, and sleep, were unavailable in the dataset, the findings should be interpreted as observational associations rather than causal effects.

### 2.4 Comparative analysis of BMI differences among different groups

The results indicate a significant difference in BMI between Han and Yi students (*t* = 31.942, *p* < 0.001), with Han students having a higher average BMI (*M* = 17.07) than Yi students (*M* = 16.90). Consistent with the analytic sample definition described in the Methods section, only the Han and Yi groups were included in the ethnicity-based analysis because sample sizes for other ethnic groups were limited, thereby ensuring statistical robustness and reliability. Regarding gender, the average BMI for males (*M* = 17.18) was significantly higher than that for females (*M* = 16.85), with a statistically significant difference (*t* = 131.733, *p* < 0.001). Within age groups, BMI showed an increasing trend with age, with the 7-year-old group having the lowest average BMI (*M* = 16.17) and the 12-year-old group having the highest (*M* = 18.08), and significant differences in BMI were observed across all age groups (*F* = 566.065, *p* < 0.001). These findings suggest that there are significant differences in BMI among students of different ethnicities, genders, and ages, with BMI generally increasing with age, and males having a higher BMI than females (see Table 1).

**Table 1:** Comparative analysis of BMI differences among different groups.

Variable	Category	*M* ± SD	Statistic	*p*	95% CI
Ethnicity	Han (*n* = 24,472)	17.07 ± 2.68	*t* = 31.942**	<0.001	0.11–0.23
	Yi (*n* = 11,620)	16.90 ± 2.69			
Gender	Male (*n* = 17,752)	17.18 ± 2.77	*t* = 131.733**	<0.001	0.27–0.39
	Female (*n* = 18,340)	16.85 ± 2.58			
Age	7 years old (*n* = 6,678)	16.17 ± 2.13	*F* = 566.065**	<0.001	–
	8 years old (*n* = 5,698)	17.05 ± 2.84			
	9 years old (*n* = 5,334)	17.86 ± 2.75			
	10 years old (*n* = 7,336)	16.24 ± 2.30			
	11 years old (*n* = 5,964)	17.21 ± 2.75			
	12 years old (*n* = 5,082)	18.08 ± 2.79			

Ethnicity and gender comparisons were performed using independent-samples *t*-tests; age-group comparison was performed using one-way ANOVA. Levene’s test was used to assess homogeneity of variance. Bonferroni correction was applied for post hoc pairwise comparisons among age groups when applicable. For two-group comparisons, 95% CIs refer to the mean difference in BMI. **p < 0.01.

### 2.5 Correlation matrix

Pearson correlation analysis showed that BMI was negatively correlated with 50-meter run time (*r* = −0.266, 95% CI: −0.275, −0.256) and lung capacity (*r* = −0.186, 95% CI: −0.196, −0.176). BMI also showed a weak negative correlation with the sit-and-reach test (*r* = −0.082, 95% CI: −0.092, −0.072) and one-minute sit-up performance (*r* = −0.153, 95% CI: −0.163, −0.143), and weak positive correlations with one-minute jump rope (*r* = 0.028, 95% CI: 0.018, 0.038) and 50-meter shuttle run (*r* = 0.047, 95% CI: 0.037, 0.057). Overall, these coefficients indicate weak to small-magnitude associations rather than strong relationships, despite their statistical significance in this large sample. Results were materially unchanged after FDR correction for multiple testing (see Table 2).

**Table 2:** Correlation matrix.

			BMI	50-meter run	Lung capacity	Sit-and-reach test	One-minute jump rope	One-minute sit-up	50-meter shuttle run
	M	SD	1	2	3	4	5	6	7
BMI	17.012	2.681	1						
50-meter run	10.342	1.149	−0.266**	1					
Lung capacity	1778.82	659.282	−0.186**	0.218**	1				
Sit-and-reach test	8.972	2.31	−0.082**	0.019**	−0.088**	1			
One-minute jump rope	86.638	22.068	0.028**	−0.191**	0.149**	0.090**	1		
One-minute sit-up	34.396	7.068	−0.153**	0.013*	0.339**	0.107**	0.214**	1	
50-meter shuttle run	134.041	34.945	0.047**	0.286**	0.063**	−0.130**	−0.070**	−0.259**	1

BMI correlations with key physical fitness indicators were as follows: 50-meter run, *r* = −0.266, 95% CI −0.275, −0.256; lung capacity, *r* = −0.186, 95% CI −0.196, −0.176; sit-and-reach test, *r* = −0.082, 95% CI −0.092,−0.072; one-minute jump rope, *r* = 0.028, 95% CI 0.018, 0.038; one-minute sit-up, *r* = −0.153, 95% CI −0.163, −0.143; and 50-meter shuttle run, *r* = 0.047, 95% CI 0.037, 0.057. Given the number of correlation tests, false discovery rate (FDR) correction was considered for multiple testing; the main findings remained materially unchanged after correction. *p < 0.05, **p < 0.01, ***p < 0.001.

### 2.6 Hierarchical regression analysis

Table 3 shows a progressive increase in the explanatory power of the hierarchical regression models. The *R*^2^ value increased from 0.009 in Model 1 to 0.041 in Model 2 and further to 0.110 in Model 3, with a significant incremental contribution in explained variance after the inclusion of physical fitness indicators and interaction terms (Delta *R*^2^ = 0.069, *p* < 0.001 for Model 3). However, the final model explained only 11.0% of the variance in BMI, indicating that the overall explanatory power of the model was modest and that most BMI variability remained unexplained by the variables included in the present analysis. This suggests that, although statistically informative, the model has limited practical value for predicting BMI at the individual level.

**Table 3:** Hierarchical regression analysis.

Predictors	Hierarchical level 1	Hierarchical level 2	Hierarchical level 3	VIF
Constant	18.507** (83.881)	24.097** (43.901)	15.732** (3.741)	
Age	0.036* (1.987)	0.025 (1.412)	0.563 (1.665)	1.42
Ethnicity	−0.047 (−0.806)	−0.041 (−0.710)	5.695** (5.236)	1.18
Gender	−0.518** (−9.833)	−0.314** (−5.810)	−3.892** (−3.782)	1.21
Lung capacity		0.000* (2.315)	0.001** (2.686)	2.07
50-meter run		−0.418** (−9.539) (−0.179)	−0.197 (−0.557) (−0.084)	1.93
Sit-and-reach test		−0.133** (−12.314) (−0.115)	−0.120 (−1.399) (−0.103)	1.88
One-minute jump rope		−0.008** (−7.400) (−0.066)	−0.054** (−6.534) (−0.445)	1.76
One-minute sit-up		−0.015** (−3.591) (−0.040)	0.193** (5.624)(0.509)	1.69
50-meter shuttle run		0.002** (3.042) (0.026)	0.022** (3.244) (0.287)	1.65
Gender × lung capacity			0.000** (3.173) (0.123)	2.31
Gender × 50-meter run			0.556** (6.481) (0.238)	2.27
Gender × sit-and-reach test			−0.289** (−13.290) (−0.249)	2.19
Gender × one-minute jump rope			0.023** (11.466) (0.189)	2.06
Gender × 50-meter shuttle run			−0.009** (−5.607) (−0.117)	2.14
Ethnicity × lung capacity			−0.001** (−6.885) (−0.246)	2.58
Ethnicity × 50-meter run			−0.372** (−3.936) (−0.160)	2.36
Ethnicity × sit-and-reach test			0.370** (15.169) (0.319)	2.41
Ethnicity × one-minute jump rope			0.009** (4.130) (0.074)	2.22
Ethnicity × one-minute sit-up			−0.134** (−14.483) (−0.353)	2.47
Ethnicity × 50-meter shuttle run			−0.009** (−4.753) (−0.117)	2.33
*R* ^2^	0.009	0.041	0.11	
Adjusted *R*^2^	0.009	0.04	0.107	
*F* value	33.997***	51.555***	49.615***	
△*R*^2^	0.009	0.032	0.069	
△*F* value	33.997***	59.784***	46.702***	

Values shown are unstandardized regression coefficients (*B*); *t* values are presented in parentheses. *p < 0.05, **p < 0.01, ***p < 0.001.

Model 1 included demographic variables; Model 2 additionally included physical fitness indicators; Model 3 additionally included interaction terms.

Because some predictors (e.g., lung capacity) were measured using large numerical units, some coefficients were very small in magnitude and should be interpreted with reference to their original measurement scales. Statistical significance should therefore be considered together with effect size and practical relevance.

In Model 1, gender was significantly associated with BMI, whereas the effect of age was comparatively small. In Model 2, several physical fitness indicators, including lung capacity, 50-meter run, sit-and-reach test, one-minute jump rope, one-minute sit-up, and 50-meter shuttle run, were significantly associated with BMI after adjustment for demographic variables. In Model 3, the inclusion of individual-level interaction terms based on gender and ethnicity further improved model fit, and multiple interaction terms remained statistically significant, suggesting that the associations between physical fitness indicators and BMI differed by gender and ethnicity. Nevertheless, these variables should be interpreted as contributing to only a limited proportion of BMI variation rather than as providing a comprehensive explanation of BMI.

It should be noted that some coefficients, particularly those for lung capacity and several interaction terms, were numerically very small. This is likely related to the original measurement scales of these variables and, to some extent, the large sample size, which increases the likelihood of detecting small effects. Therefore, the regression findings should be interpreted not only on the basis of statistical significance, but also in terms of coefficient magnitude, incremental explained variance, and practical relevance. In particular, statistically significant coefficients with very small magnitudes should not be overstated as evidence of meaningful clinical or behavioral effects.

**Figure 1:**
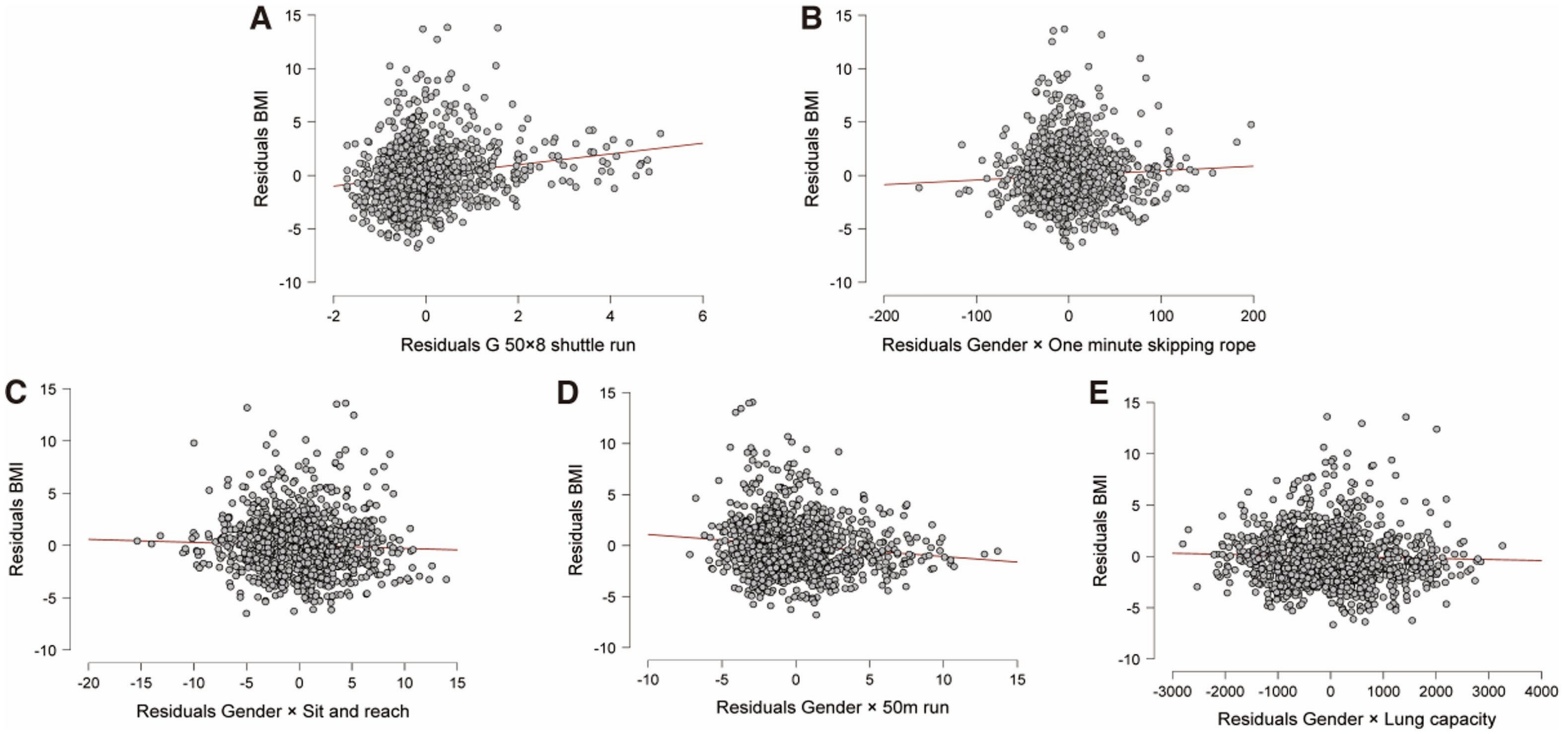
Relationship between Gender × physical fitness indicators and BMI residuals.

**Figure 2:**
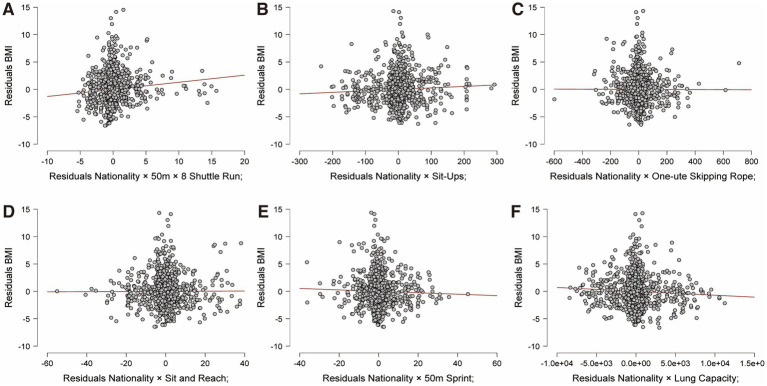
Relationship between Ethnicity/Nationality × physical fitness indicators and BMI residuals.

Model diagnostics indicated that the residuals were approximately normally distributed, with no evidence of substantial heteroscedasticity. All variance inflation factors (VIFs) were below 5, indicating no serious multicollinearity. To facilitate interpretation, Figures 1, 2 present the main-effect trends and interaction patterns from Models 2 and 3, respectively, thereby illustrating the direction and relative magnitude of the observed associations.

## 3 Discussion

This study provides an in-depth analysis of the association between physical fitness and BMI among primary school students in Sichuan Province, and from a developmental and contextual perspective, reveals significant differences in BMI across gender, ethnic, and age groups as well as interaction patterns among these factors. Firstly, the study shows that boys have a significantly higher BMI than girls, consistent with the findings of Liu et al. (10). This finding is in line with previous research suggesting that gender differences are associated with variation in children’s BMI (11), and may be related to differences in muscle mass and bone density between boys and girls. However, this study further suggests that gender is associated not only with BMI itself but also with differences in the associations between BMI and other physical health indicators. For example, significant interactions between gender and physical fitness indicators such as lung capacity and 50-meter run time suggest that these associations may differ by gender and may warrant further investigation in subgroup-specific research and school health planning.

Regarding ethnic differences, the study found that Han students had a significantly higher BMI than Yi students, consistent with the results of Gao et al. (12). These findings apply only to the Han and Yi groups included in the ethnicity-based analysis and should not be generalized to other ethnic populations because of the limited sample sizes for other groups. Yu et al. noted that differences in socioeconomic background, dietary habits, and lifestyle between Han and Yi students may be associated with the observed BMI differences (13). These ethnic differences may be partly related to differences in living environment, lifestyle, and access to resources; however, these factors were not measured directly in the present study.

The analysis of age groups shows that BMI significantly increases with age, particularly reaching the highest value in the 12-year-old group. This trend is consistent with the findings of Cheng et al. (14), who reported that rapid growth in weight and height during puberty is accompanied by an increase in BMI. However, the results also suggest that the upward trend in BMI may be related to changes in lifestyle. As children grow older, their lifestyles may become more sedentary, and reduced physical activity may be associated with higher body weight. In addition, this pattern may reflect the combined influence of developmental changes in body composition, school workload, screen exposure, and age-related differences in daily movement opportunities, although these factors were not directly measured in the present study. García-Hermoso et al. (15) believe that weight management in adolescents is closely related to exercise habits. Therefore, at different stages of children’s growth, attention should be paid to the balance between weight management and physical health, and future studies may further examine whether age-appropriate health education and exercise programs are associated with more favorable BMI-related outcomes.

The correlation matrix analysis further reveals statistically significant relationships between BMI and various physical health indicators. However, the observed correlation coefficients were generally small in magnitude, indicating weak associations rather than strong relationships. For example, BMI showed a negative correlation with 50-meter run performance and lung capacity, consistent with the research of Pan et al. (16). These findings suggest that students with higher BMI values tended, on average, to show less favorable athletic and cardiorespiratory performance, although the practical magnitude of these associations was limited. Additionally, BMI was negatively correlated with flexibility (such as the sit-and-reach test), but this association was also weak. Although the correlations between BMI and strength/endurance indicators (such as one-minute jump rope and sit-ups) were statistically significant, their effect sizes were small and should be interpreted cautiously in terms of practical importance. One possible explanation is that BMI is a relatively crude anthropometric indicator and does not distinguish fat mass from lean mass, whereas physical fitness is multidimensional and may be influenced by training habits, maturation status, motivation during testing, and other unmeasured behavioral or environmental factors. Moreover, in a large sample, even modest associations may reach statistical significance; therefore, statistical significance should not be interpreted as evidence of strong practical effects. From a practical perspective, the present findings should be interpreted with caution. Although multiple associations reached statistical significance, most effect sizes were small, and the final regression model explained only a limited proportion of BMI variance. This indicates that the observed relationships are more useful for identifying broad population-level patterns than for supporting individual-level clinical judgment or precise risk prediction. Accordingly, these findings may help inform school health surveillance and the design of region-specific health promotion strategies, but they should not be taken to imply that any single physical fitness indicator has a strong or clinically meaningful association with BMI in isolation.

The hierarchical regression analysis suggests that gender, ethnicity, age, and several physical fitness indicators were associated with BMI, and that some of these associations varied according to gender and ethnicity. For example, significant interaction terms between gender and physical fitness indicators such as lung capacity, 50-meter run performance, and sit-and-reach test suggest that the associations between these indicators and BMI may differ between boys and girls. Consistent with the findings of Gao et al. (17), the present study supports a possible moderating role of gender in the relationship between physical fitness and BMI. Similarly, significant interaction terms involving ethnicity suggest that the associations between selected physical fitness indicators and BMI may vary across ethnic groups, which is broadly consistent with the findings of Liu et al. (18). However, because the explanatory power of the final model was limited (*R*^2^ = 0.110), these findings should be interpreted as partial and modest associations rather than as evidence of substantial effects or of the major determinants underlying BMI variation. This relatively low explained variance also suggests that BMI is likely shaped by multiple factors beyond the variables included here, such as diet, family environment, habitual physical activity, sleep, and broader social context, which may dilute the independent contribution of any single physical fitness indicator in baseline observational analyses.

Although this study provides new insights into the relationship between physical health and BMI among primary school students in Sichuan Province, there are some limitations. First, the study uses a cross-sectional design, which does not allow for the inference of causal relationships. Therefore, future research should consider using longitudinal data to further explore the causal relationship between BMI and physical health indicators. Second, the study mainly focuses on children in Sichuan Province; although the sample has certain representativeness, the results may not be fully generalizable to other regions. To verify and extend the findings of this study, future research should expand the sample scope to include children from more diverse geographical, ethnic, and socioeconomic backgrounds.

Additionally, important confounding variables such as diet, socioeconomic status, physical activity, and sleep were not adjusted for. This omission may have introduced substantial residual confounding and may have affected both the magnitude and, potentially, the direction of the observed associations, in addition to partly explaining the relatively low explanatory power of the final regression model. Therefore, the findings should be interpreted cautiously as associative rather than causal, and should not be taken as providing precise estimates of the independent relationships between physical fitness indicators and BMI. Since the final model explained only 11.0% of the variance in BMI, a substantial proportion of BMI variability remains unexplained. Moreover, given the large sample size, statistical significance should not be interpreted as equivalent to strong practical significance. Future research should incorporate a broader range of behavioral, familial, environmental, and socioeconomic variables to improve model fit and enhance the reliability of the findings. In addition, although the sample was drawn from multiple schools and regions, the present analysis focused on hierarchical multiple regression at the individual level rather than multilevel modeling of clustered data, and no school- or region-level random effects were estimated. Future studies may consider multilevel or mixed-effects models to further account for potential school- or region-level clustering effects. Taken together, these limitations suggest that the current findings are best viewed as preliminary baseline associations rather than definitive estimates of the determinants of BMI.

## Data Availability

The data used in this study can be obtained by contacting Wennan Zhao (Email: zhaown@ysu.edu.cn) for further academic research.
